# A bidirectional causal relationship study between mental disorders and male and female infertility

**DOI:** 10.3389/fpsyt.2024.1378224

**Published:** 2024-04-18

**Authors:** Xiangyu Chen, Xuexue Hao, Lijun Xie, Xiaoqiang Liu

**Affiliations:** Department of Urology, Tianjin Medical University General Hospital, Tianjin, China

**Keywords:** mental disorders, male infertility, female infertility, Mendelian randomization, genome-wide association study (GWAS)

## Abstract

**Background:**

The relation between mental disorders (MDs) and infertility can be reciprocal. But exactly which MD affects infertility remains controversial. Our aim was to use Mendelian randomization (MR) to explore bidirectional causality between 15 MDs and male infertility and female infertility.

**Methods:**

The data of MDs, male infertility, and female infertility were derived from published genome-wide association studies (GWAS). The inverse variance weighted method was considered to be the main analytical approach. Sensitivity analysis was performed using MR-Egger, Cochran’s Q, radial MR, and MR-PRESSO tests.

**Results:**

Our results found that mood disorders (OR, 1.4497; 95% CI, 1.0093 – 2.0823; P = 0.0444) and attention deficit hyperactivity disorder (OR, 1.3921; 95% CI, 1.0943 – 1.7709; P = 0.0071) were positively correlated with male infertility, but obsessive-compulsive disorder (OR, 0.8208; 95% CI, 0.7146 – 0.9429; P = 0.0052) was negatively associated with male infertility. For females, anorexia nervosa (OR, 1.0898; 95% CI, 1.0070 – 1.1794; P = 0.0329), attention deficit hyperactivity disorder (OR, 1.1013; 95% CI, 1.0041 – 1.2079; P = 0.0406), and major depressive disorder (OR, 1.1423; 95% CI, 1.0213 – 1.2778; P = 0.0199) increased risk of infertility. In reverse relationship, female infertility increased the incidence of bipolar disorder (OR, 1.0009; 95% CI, 1.0001 – 1.0017; P = 0.0281).

**Conclusion:**

We demonstrated the association between five MDs and male or female infertility. Female infertility was also found to be associated with an increased risk of one MD. We look forward to better designed epidemiological studies to support our results.

## Introduction

Infertility is defined as the inability to conceive after at least 12 months of regular unprotected intercourse (1). Infertility is a major health problem worldwide, affecting an estimated 8-12% of couples of reproductive age (2). Between 1990 and 2017, the age-standardized prevalence rate of infertility increased by 0.291% per year in men and 0.370% per year in women worldwide (3). Men were found to be the sole cause for 20–30% of infertility cases but contributed to 50% of cases overall (4). Separate explanations of male and female infertility are not appropriate for couples with infertility; therefore, a parallel assessment of both partners is always needed.

Over 25% of people worldwide suffer from various psychiatric disorders that are the primary causes of disability (5). Mental disorders (MDs), one of the top ten leading causes of disease burden globally, are particularly burdensome in high- and upper-middle income countries (6). MDs occur in diverse forms and present in different ways; however, they are generally characterized by a combination of abnormal thoughts, perceptions, behaviors, emotions, and relationships with others (7). MDs include depression, anxiety, schizophrenia, bipolar disorder, mood disorders and other psychotic disorders, Alzheimer’s disease, and Parkinson’s disease, among others (8). There are significant sex differences in the prevalence of MDs, with depression, bipolar disorder, and anxiety occurring more frequently in women, while men tend to experience more apathy, poverty of speech and thought, and social withdrawal (9). The relationship between MDs and infertility has been controversial for several years. Several studies have investigated the relationship between psychological symptoms before and during assisted reproductive treatment and subsequent pregnancy rates, with conflicting results. Some studies have suggested that the more distressed the women were before and during treatment, the lower the pregnancy rate, while others have not (10–12). Conversely, many infertile couples experience various problems, especially significant changes in the quality of their sex life and mental state (13, 14). Previous studies have shown that infertile men have a higher prevalence of sexual dysfunction than the general population, and suffer from MDs due to self-inflicted, conjugal, and social stressors (15–17).

As mentioned previously, the relationship between MDs and infertility is complex, multifactorial, and bidirectional. Moreover, previous observational and randomized controlled studies have also been affected by confounding factors, which have resulted in biased results. Thus, it remains controversial whether MDs and infertility interact with each other. The aim of our study was to explore the bidirectional causality between different MDs and infertility in men and women using published genome-wide association study (GWAS) data with two-sample Mendelian randomization (TSMR) and multivariate Mendelian randomization (MVMR) methods. By applying a bidirectional Mendelian randomization (MR) approach, we can explore whether MDs casually affects infertility risk and we can also examine whether the genetic predisposition to infertility causally influences the MDs. Based on above, we sought to clarify the role of MDs and infertility in their possible association, providing a basis for clinicians to develop effective prevention, diagnosis, and treatment strategies. MR is an epidemiological genetics method based on Mendel’s law that is used to estimate the causal relationships between exposures and outcomes. In epidemiological studies, confounding factors significantly impede the establishment of a causal relationship between exposure and outcome. The MR method, in theory, effectively circumvents the influence of confounding factors and eliminates the interference caused by reverse causality. Currently, the MR method is extensively employed to assess the causal association between traits and diseases as well as among different diseases.

## Methods

### Data collection

Genetic data for the MDs phenotype in this study were obtained from a large-scale GWAS meta-analysis, specifically of European populations (**Table 1**). Data on anxiety, depression, epilepsy, insomnia, and stroke were obtained from the Medical Research Council-Integrative Epidemiology Unit (MRC-IEU). The Alzheimer’s Disease (AD) data were sourced from the Alzheimer’s Disease Genetics Consortium (ADGC). The anorexia nervosa (AN) data were obtained from the Psychiatric Genomics Consortium-Eating Disorders (PGC-ED) working group. Autism spectrum disorder (ASD) data were sourced from the Integrative Psychiatric Research-Psychiatric Genomics Consortium (iPSYCH-PGC). Data on bipolar disorder were sourced from the Neale Lab. Data on major depressive disorder were sourced from the PGC. Data on mood and obsessive-compulsive disorders were sourced from the FinnGen consortium. The Parkinson’s disease (PD) data were sourced from the International Parkinson’s Disease Genomics Consortium. Schizophrenia data were sourced from the Schizophrenia Working Group of the PGC.

**Table 1:** Detailed information about data sources of mental disorders and infertility.

Traits	GWAS ID	Sample size	SNPs	Year	Pubmed ID (or URL)	P value	F-statistics
Alzheimer’s disease	ieu-b-2	63,926	10,528,610	2019	30820047	5×10^-6^	68.9583
*Anorexia nervosa*	ieu-a-1186	14,477	10,641,224	2017	28494655	5×10^-6^	23.5234
*Anxiety*	ukb-b-17243	462,933	9,851,867	2018	http://app.mrbase.org	5×10^-6^	22.6156
*Attention deficit hyperactivity disorder*	ebi-a-GCST005362	32,102	7,414,807	2017	29325848	5×10^-6^	23.7360
*Autism spectrum disorder*	ieu-a-1185	46,351	9,112,386	2017	http://app.mrbase.org	5×10^-6^	24.1026
*Bipolar disorder*	ukb-a-83	337,159	10,894,596	2017	http://app.mrbase.org	5×10^-6^	23.3285
*Depression*	ukb-b-12064	462,933	9,851,867	2018	http://app.mrbase.org	5×10^-6^	23.5320
*Epilepsy*	ukb-b-16309	462,933	9,851,867	2018	http://app.mrbase.org	5×10^-6^	23.9022
*Insomnia*	ukb-b-3957	462,341	9,851,867	2018	http://app.mrbase.org	5×10^-6^	29.3238
*Major depressive disorder*	ieu-a-1188	173,005	13,554,550	2018	29700475	5×10^-6^	24.9650
*Mood disorders*	finn-b-KRA_PSY_MOOD	218,792	16,380,466	2021	http://app.mrbase.org	5×10^-6^	23.1883
*Obsessive-compulsive disorder*	finn-b-F5_OCD	199,169	16,380,384	2021	http://app.mrbase.org	5×10^-6^	22.8906
*Parkinson’s disease*	ieu-b-7	482,730	17,891,936	2019	http://app.mrbase.org	5×10^-6^	38.6477
*Schizophrenia*	ieu-b-42	77,096	15,358,497	2014	25056061	5×10^-6^	28.9780
*Stroke*	ukb-b-6358	462,933	9,851,867	2018	http://app.mrbase.org	5×10^-6^	23.0032
*Alcoholic drinks per week*	ieu-b-73	335,394	11,887,865	2019	30643251	NA	NA
*Cigarettes per day*	ieu-b-25	337,334	11,913,712	2019	30643251	NA	NA
*Male infertility*	finn-b-N14_MALEINFERT	73,479	16,377,329	2021	http://app.mrbase.org	5×10^-6^	NA
*Female infertility*	finn-b-N14_FEMALEINFERT	75,450	16,377,038	2021	http://app.mrbase.org	5×10^-6^	NA

NA, Not available.

Data on male infertility (680 cases and 72,799 controls) and female infertility (6,481 cases and 68,969 controls) were obtained from the FinnGen Consortium.

### Instrumental variables selection

**Figure 1:**
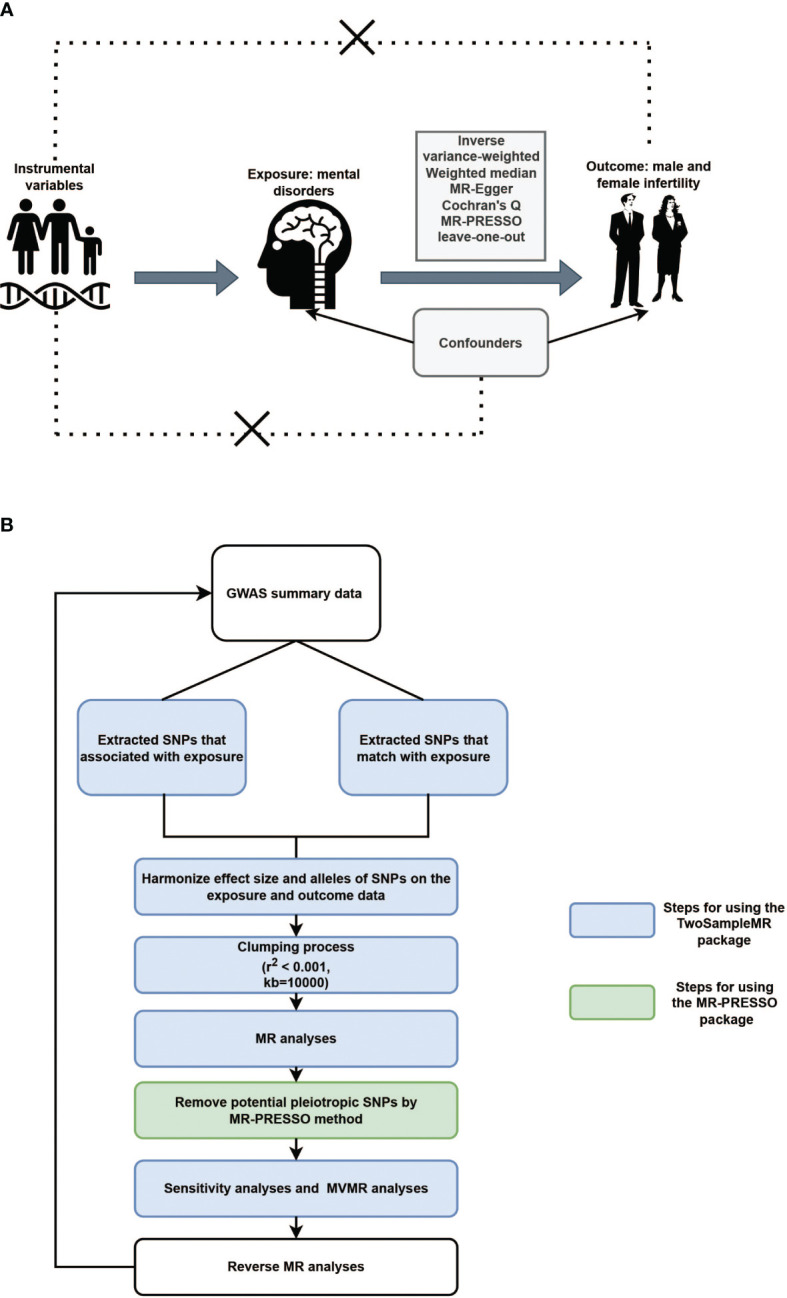
**(A)**. Illustrative diagram of Mendelian randomization assumptions. **(B)**. Flowchart of Mendelian randomization analytical processes.

Three fundamental assumptions must be met for a convincing MR study to be conducted: (1) the genetic instruments are assumed to have direct associations with exposure; (2) the genetic instruments are assumed to be unrelated to the outcome and independent of any known or unknown confounding factors; and (3) the effects of instrumental variables (IVs) on the outcomes are exclusively mediated by the exposures of interest (**Figure 1A**). The criteria used for the selection of single-nucleotide polymorphisms (SNPs) were as follows: (1) SNPs that were associated with MDs and infertility were extracted with a GWAS threshold (p<5×10^-6^); (2) SNPs in linkage disequilibrium (LD) were identified and excluded using the LD clumping method (r^2^ < 0.001, kb=10000). We also searched the PhenoScanner website (http://www.phenoscanner.medschl.cam.ac.uk/information/) for the IVs identified for inclusion in the study. If SNPs associated with the outcomes were present, they were excluded from the MR analysis. Whether the selected IVs had weak IVs bias was assessed by calculating the F-statistic. If F > 10, this indicates that there is no weak IVs bias.

### Statistical analysis

Inverse variance-weighted (IVW), weighted median (WM), and MR-Egger tests were applied for the TSMR analysis. When the IVs satisfy all three assumptions cited above, the IVW method can provide consistent estimates of the causal effect of exposure and is considered the strongest MR method. In the sensitivity analysis, Cochran’s Q test was used to examine heterogeneity. When heterogeneity was observed, a random-effects IVW model was used. MR-Egger was used to estimate horizontal pleiotropy according to its intercept, ensuring that genetic variation was independently associated with exposure and outcomes. Additionally, we employed the MR pleiotropy residual sum and outlier (MR-PRESSO) and radial MR tests to identify pleiotropic SNPs, eliminate outliers from horizontal pleiotropy, and determine whether there were statistically significant variations in the causal estimates prior to and following outlier correction using the distortion test. Finally, the stability of the TSMR results was determined by performing a “leave-one-out” analysis of the data, sequentially excluding one SNP to estimate whether a single SNP was driving or biasing the results. **Figure 1B** illustrated the flowchart of MR analytical processes.

Statistical analysis was performed using R Software (version 4.3.2), through TwoSampleMR (0.5.5) (18) and MR-PRESSO (1.0) (19) packages, and P < 0.05 was statistically significant for evidence of potential causal effect.

## Results

### Positive causal association analysis for MDs and infertility

**Figure 2:**
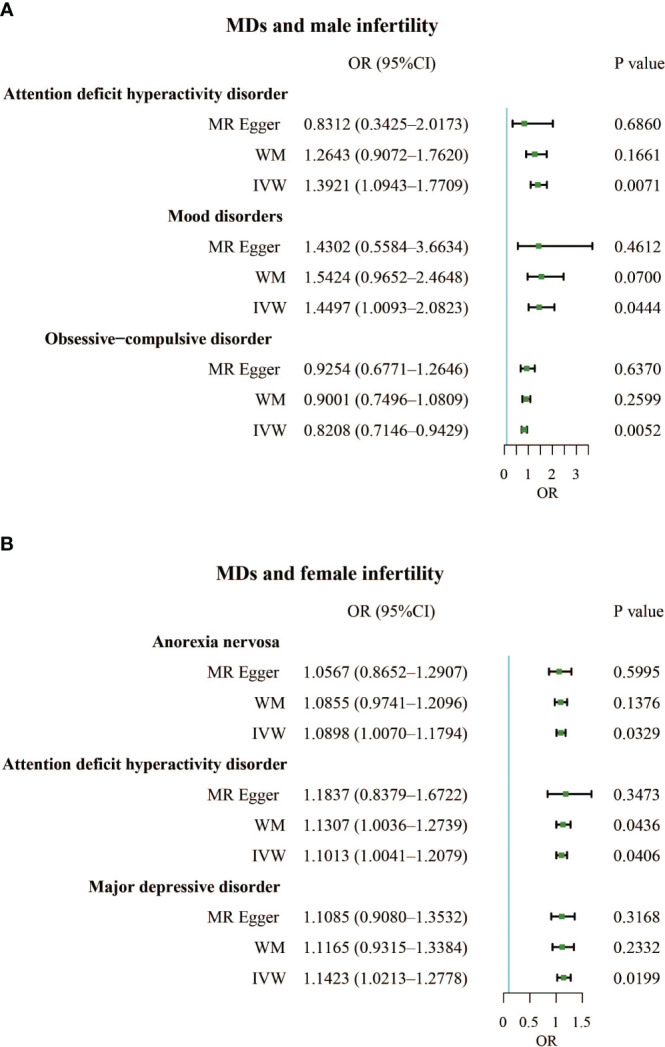
**(A)**. Forest plot of the causal associations between attention deficit hyperactivity disorder, mood disorders, and obsessive-compulsive disorder and male infertility. **(B)**. Forest plot of the causal associations between anorexia nervosa, attention deficit hyperactivity disorder, and major depressive disorder and female infertility.

The F-statistics for all exposures were higher than 10, indicating a low risk of weak instrumental bias. The results of the TSMR analysis showed a positive correlation between mood disorders and the incidence of male infertility (Odds Ratio [OR], 1.4497; 95% Confidence Interval [CI], 1.0093 – 2.0823; P = 0.0444), indicating that mood disorders elevated the incidence of male infertility. Additionally, attention-deficit hyperactivity disorder was a risk factor for male infertility (OR, 1.3921; 95% CI, 1.0943 – 1.7709; P = 0.0071). However, obsessive-compulsive disorder had a protective effect against male infertility (OR, 0.8208; 95% CI, 0.7146 – 0.9429; P = 0.0052) (**Figure 2A**; **Supplementary Table 1**). We also identified three MDs that contributed to female infertility, namely anorexia nervosa (OR, 1.0898; 95% CI, 1.0070 – 1.1794; P = 0.0329), attention deficit hyperactivity disorder (OR, 1.1013; 95% CI, 1.0041 – 1.2079; P = 0.0406), and major depressive disorder (OR, 1.1423; 95% CI, 1.0213 – 1.2778; P = 0.0199) (**Figure 2B**; **Supplementary Table 1**).

In addition, a sensitivity analysis was conducted to verify the precision of the findings. In terms of male and female infertility, neither the IVW test nor the MR-Egger test revealed any heterogeneity. Furthermore, the MR-Egger intercept test detected horizontal pleiotropy in the relationship between schizophrenia and male infertility (P = 0.0377). However, MR-PRESSO did not detect pleiotropy. The MR-Egger and MR-PRESSO tests did not reveal horizontal pleiotropy in the causal association results between the other MDs and male (or female) infertility (**Supplementary Table 3**). Based on the leave-one-out analysis, no single SNP had a large impact on the robustness of the results after the individual removal tests (**Supplementary Figure 1**; **Supplementary Figure 2**).

### Reverse causal association analysis for infertility and MDs

**Figure 3:**
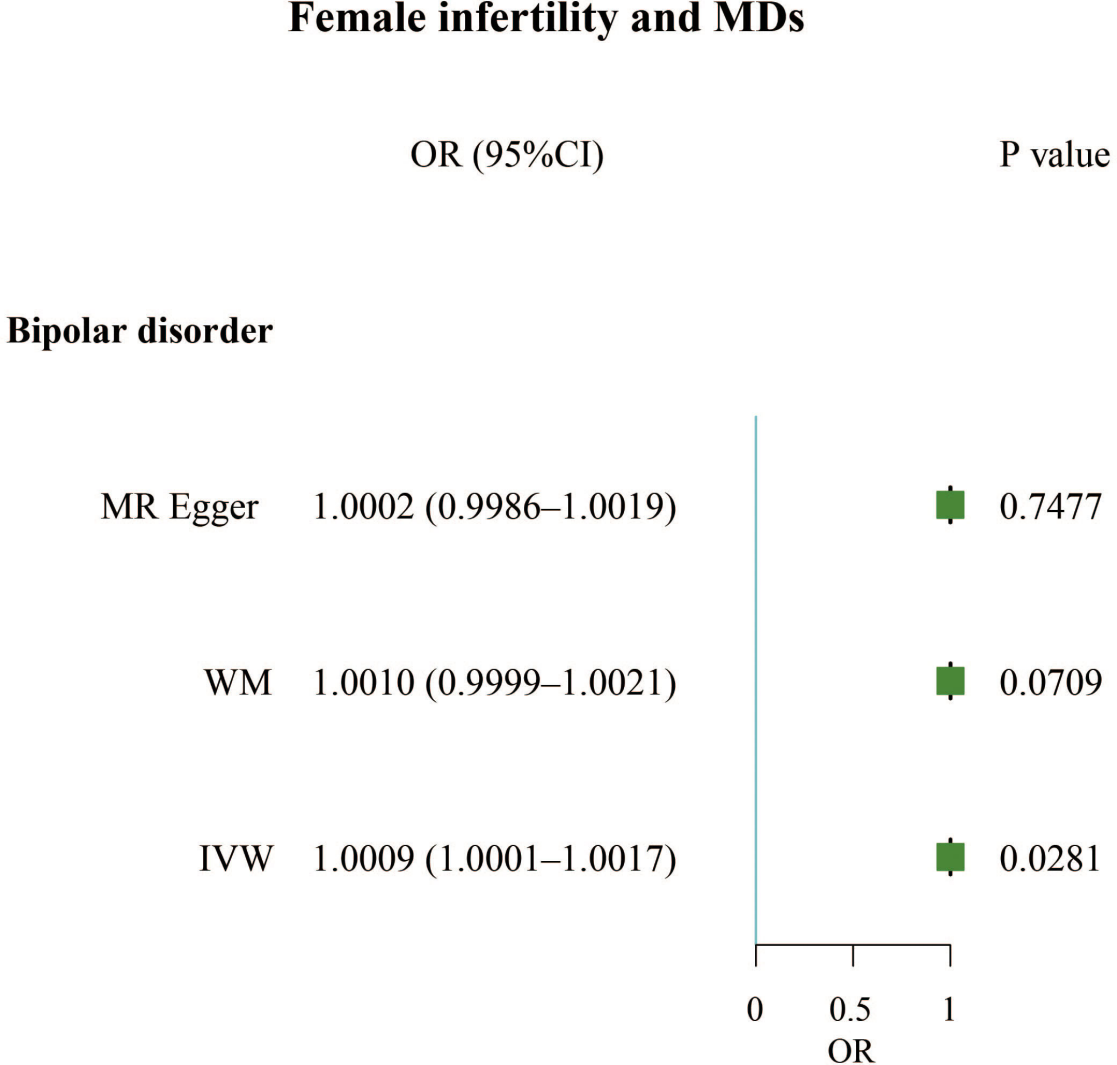
Forest plot of the causal associations between female infertility and bipolar disorder.

The F-statistics for all exposures were higher than 10, indicating a low risk of weak instrumental bias. We found that infertility led to a slightly increased incidence of bipolar disorder in women (OR, 1.0009; 95% CI, 1.0001 – 1.0017; P = 0.0281). However, male infertility has not been found to cause MDs (**Figure 3**; **Supplementary Table 2**).

In the sensitivity analysis, the IVW and MR-Egger tests revealed heterogeneity in the results of female infertility and obsessive-compulsive disorder, female infertility and insomnia, and male infertility and schizophrenia. Although the MR-Egger intercept test did not detect horizontal pleiotropy in any of the results, the MR-PRESSO test found horizontal pleiotropy in the results of female infertility and obsessive-compulsive disorder, female infertility and insomnia, and male infertility and schizophrenia (**Supplementary Table 4**). Except for the results of female infertility and obsessive-compulsive disorder that had no significant outliers, the results of female infertility and insomnia (excluding rs111992780), and male infertility and schizophrenia (excluding rs114146352 and rs55841791) did not affect the original causality when outliers were excluded (**Supplementary Figure 3**; **Table 2**). Based on the leave-one-out analysis, no single SNP had a large impact on the robustness of the results after the individual removal tests (**Supplementary Figure 4**).

**Table 2:** Radial MR method to test for heterogeneity and horizontal pleiotropy in IVW and MR Egger.

		Estimate	SE	t value	P value
Male infertility and schizophrenia	Radial IVW				
	Effect (1st)	-0.0332	0.0206	-1.6108	0.1072
	Iterative	-0.0333	0.0206	-1.6117	0.1070
	Exact (FE)	-0.0378	0.0118	-3.1981	0.0013
	Exact (RE)	-0.0345	0.0211	-1.6388	0.1356
	Q-Statistic for heterogeneity	NA	NA	28.7938	0.0007
	Radial MR-Egger				
	Intercept	-1.3215	2.7334	-0.4834	0.6416
	Wj	0.0138	0.0996	0.1387	0.8931
	Q-Statistic for heterogeneity	NA	NA	27.9763	0.0004
Female infertility and insomnia	Radial IVW				
	Effect (1st)	0.0046	0.0077	0.5999	0.5485
	Iterative	0.0046	0.0077	0.5997	0.5486
	Exact (FE)	0.0050	0.0048	1.0382	0.2991
	Exact (RE)	0.0047	0.0069	0.6914	0.5067
	Q-Statistic for heterogeneity	NA	NA	23.0848	0.0060
	Radial MR-Egger				
	Intercept	0.6515	2.5886	0.2517	0.8076
	Wj	-0.0051	0.0396	-0.1289	0.9006
	Q-Statistic for heterogeneity	NA	NA	22.9034	0.0034

NA, Not available.

### Multivariate Mendelian randomization

To examine whether the role of MDs in infertility was mediated by smoking and alcohol consumption, we performed MVMR analyses. After adjusting for attention-deficit hyperactivity disorder with cigarettes per day and alcoholic drinks per week, attention-deficit hyperactivity disorder remained positively associated with male infertility (OR, 1.8497; 95% CI, 1.1195 – 3.0563; P = 0.0164). Additionally, weekly alcohol consumption in infertile women slightly reduced the risk of bipolar disorder (OR, 0.9971; 95% CI, 0.9945 – 0.9998; P = 0.0323) (**Supplementary Figure 5**).

## Discussion

This study used TSMR, MVMR, and radial MR to explore the bidirectional causal relationships between 15 MDs and infertility. We demonstrated that mood disorders and attention deficit hyperactivity disorder increased the risk of male infertility; obsessive-compulsive disorder decreased the incidence of male infertility. However, male infertility has not been found to cause any MDs. Anorexia nervosa and attention deficit hyperactivity disorder were positively correlated with female infertility, while female infertility contributed to bipolar disorder.

Stress is a constant threat to homeostasis and is represented by different extrinsic and intrinsic stimuli (20). As a result of increasingly negative socioeconomic factors such as bereavement, job insecurity, isolation, loneliness, or financial problems, short-term pressures may become long-term (21). Psychological, lifestyle, and oxidative stress increase the risk of MDs and are accompanied by a series of pathological reactions that lead to a rebalancing of the body, including pathological effects on metabolism, vascular function, tissue repair, immune function, and the nervous system (22–24). A cross-sectional study of 1,215 men showed that men with high self-reported stress scores had poorer semen quality than those with moderate stress; sperm concentration, total sperm count, and semen volume were all significantly lower (38%, 34%, and 15%, respectively) (25). With respect to the mechanism responsible for this outcome, we speculate that various stresses and concomitant psychiatric disorders activate the hypothalamic–pituitary–adrenal (HPA) axis to release arginine vasopressin and corticotropin-releasing hormone (CRH) that mediate the secretion of adrenocorticotropic hormone (ACTH) from the anterior pituitary gland, and ACTH in turn mediates the secretion of glucocorticoids from the adrenal cortex (26, 27). Glucocorticoids induce apoptosis of Leydig cells and reduce testosterone levels. A decrease in testosterone levels leads to a cascading effect on Sertoli cells and the blood-testis barrier, thereby reducing spermatogenesis (28, 29). In females, glucocorticoids cause a severe dysfunction of the hypothalamic-pituitary-ovary (HPO) axis, altering the physiological release of gonadotropin-releasing hormone (GnRH) and causing abnormal luteinizing hormone (LH) pulses (30–32). Anomalous LH pulses can directly or indirectly inhibit ovulation through their effect on the synthesis and secretion of sex steroids in the ovary (33). Both in the general and the infertile population, mental distress is associated with lower conception rates, longer menstrual cycles (≥35 d), and lower reproductive medicine outcomes, including rates of egg retrieval, fertilization, pregnancy, and live births. Mental distress also impairs ovarian reserve and contributes to a less tolerant endometrium (34–36).

A possible link between male infertility and sexual dysfunction is manifested in psychopathological disorders associated with both conditions. Erectile function is poorer in infertile men compared to fertile men and is associated with an overall psychopathological burden, especially somatization of anxiety (37, 38). A cross-sectional study found that in men with azoospermia, erectile function was negatively associated not only with psychopathological disturbances (Middlesex Hospital Questionnaire [MHQ] total and Middlesex Hospital Questionnaire-Somatized anxiety [MHQ-S] scores; P < 0.0001) but also with a less healthy phenotype (higher Chronic Disease Score [CDS]; P = 0.015) (38). In addition, men with azoospermia have a higher incidence of premature ejaculation, and a lower libido and orgasmic function than fertile men: all of these dysfunctions are associated with psychopathologic symptoms (39). MDs increase progressively with semen damage, and couples experience more depressive symptoms and somatic anxiety when they realize that sex does not lead to pregnancy (40, 41).

Psychotropic medications may also affect the reproductive function in both men and women. Hyperprolactinemia due to antipsychotics can indirectly cause hypogonadism that may adversely affect sperm parameters including sperm count and viability (42, 43). Serotonin reuptake inhibitors (SRIs) are a standard treatment for depression. The loss or absence of sexual pleasure is frequently reported during SRI treatment. Delayed ejaculation, or even the absence of ejaculation, also begins a few weeks after the start of treatment (44). A cohort study that examined women who had spent < 3 months in their attempts to conceive discovered a correlation between antidepressant use and decreased fecundability in any given menstrual cycle, regardless of the history of depression of the patient (45). Another cohort study on women attempting to conceive found that benzodiazepine use was associated with reduced fertility that may indicate more severe symptoms of anxiety or the presence of comorbidities (46). MDs are a chronic condition where discontinuation of antidepressant treatment is not an option. Changing the treatment of MDs is also difficult, and there is insufficient data on the benefits of “treatment interruption” or dose reduction in improving the sexual functioning of patients (47).

Major depressive disorder is the second most prevalent psychiatric disease among female infertility patients (48). Depression usually affects couples trying to conceive, either directly or indirectly, although there are conflicting results regarding their effects on fertility. It is unclear whether pre-morbid diagnoses of depression contribute to infertility or whether they are caused by the psychological distress of infertility and its treatment (49, 50). A cohort study indicated that male partners with active major depressive disorder were less likely to have a partner achieve conception. However, the presence of active depression was not associated with poorer birth outcomes (live births, miscarriages) in the women in this study who were not using antidepressants, but rather with a slightly increased likelihood of pregnancy (51). Our results were contrary, and we concluded that depression increased the risk of infertility in women, although subgroup analyses of whether female depressed patients were taking antidepressants or not are needed to further confirm our conclusions. Next, anorexia nervosa impairs female fertility due to ovulatory dysfunction and reduced sexual activity and is the most common cause of infertility in underweight women (52). Anorexia nervosa is a complex psychosomatic eating disorder that includes emotional and behavioral problems, such as a severely restricted food intake, excessive exercise, and extreme fear of weight gain, and primarily affects adolescent girls and young women. One study reported that 60% of women with infertility had eating disorders (53). In another study, ovulatory infertility was more frequent in patients with eating disorders (n=271, of which 111 had anorexia nervosa) compared to women without eating disorders using assisted reproductive technologies (ART) (16.2 vs. 5.6%, respectively; P < 0.001) (54). Given these facts, the American College of Obstetricians and Gynecologists (ACOG) recommends that all women with eating disorders should receive counseling and be provided with access to birth control (55). In addition, previous studies have shown that children born to infertile mothers not receiving fertility treatments are at a higher risk of being diagnosed with attention deficit hyperactivity disorder, and women have a lower absolute incidence of attention deficit hyperactivity disorder but a relatively higher hazard ratio than men (56). Our study further revealed that attention deficit hyperactivity disorder increased the risk of infertility in both men and women. In conjunction with these results, we recommend that the mental as well as reproductive health of the offspring of patients with attention deficit hyperactivity disorder be considered when assessing the fertility of these patients. Finally, there were some sex differences in the clinical features and course of bipolar disorder; a significantly higher proportion of male patients had comorbid substance dependence, whereas the prevalence of somatic comorbidities was significantly higher in female patients (57). Not only did our study demonstrate that infertility can slightly increase the risk of women developing bipolar disorder, but the MVMR analysis also found that alcoholic drinks per week appeared to reduce this risk. However, given the negative effects of alcohol on fertility, this form of relief for psychiatric disorders needs to be used with caution (58, 59).

As technological advances and options for family building through ART have developed, the need for and utilization of psychological services or psychiatric treatment has also increased (60). MDs may affect the quality of life of people facing ART and ART outcomes. Previous studies have demonstrated that alexithymia was associated with worse quality of life for women in couples or partners who began ART (61). In another study, women who were diagnosed with depression before ART treatment had a lower mean number of ART live births compared with women without a depression diagnosis (62). Therefore, it is worthwhile for clinicians to develop programs to reduce MDs in infertile patients for more ART success. Several meta-analyses have indicated that psychological interventions not only have a positive impact on pregnancy rates in couples not treated with ART (Risk Ratios [RR], 1.42; 99% CI: 1.02 – 1.96) (63), but also improve pregnancy rates in patients receiving ART treatment (RR, 1.43; 95% CI: 1.07 – 1.93) (64). Additionally, psychosocial interventions, particularly long-duration interventions, have been shown to be positively associated with pregnancy rates (65). Although psychological interventions have been found to have an impact on conception, causal language is considered necessary to use when discussing the link between MDs and conception (66). This means that the value of psychiatric or psychological interventions should not be limited to improving conception rates, but it is also important to reduce stress or psychological distress in patients (66). Additionally, patients should be encouraged to pursue individualized treatments and modalities, rather than specific treatments, to better tailor to the realities of living with infertility.

Our study has some limitations. First, the GWAS data included in our MR analyses were primarily derived from participants of European ancestry, and the applicability of these findings to other populations remains to be confirmed. Second, more relevant SNPs are required to provide more robust causality estimates. Nevertheless, this study has several strengths. First, compared with earlier observational studies, the sample sizes in our study were significantly larger, and this may have provided sufficient statistical power to determine causality. Next, we used multifactorial MR to correct for the effects of smoking and alcohol consumption on exposure, thus making our results more accurate. Finally, sensitivity analysis was conducted to minimize potential confounders and reverse causality. The relationship between mental illness and infertility is complex. Given that many patients are reluctant to discuss their suffering and few seek help, understanding the impact of psychiatric treatment on fertility is critical for clinicians treating couples trying to conceive. In the future, we look forward to better designed epidemiological studies with larger sample sizes to support our results.

## Conclusion

Our study utilized MR to reveal that two MDs were positively associated with male infertility, one MD was negatively associated with male infertility, and three MDs were positively associated with female infertility. In reverse relationship, female infertility was found to be positively correlated with one MD, but male infertility was not found to be correlated with MDs.Attention deficit hyperactivity disorder and mood disorders were risk factors for male infertility; anorexia nervosa, attention deficit hyperactivity disorder, and major depressive disorder were risk factors for female infertility. However, obsessive-compulsive disorder appeared to be a protective factor against male infertility. Additionally, we found that female infertility can lead to bipolar disorder, and MVMR analysis showed that alcoholic drinks per week can slightly alleviate this mental illness. More high-quality prospective studies are needed in the future to confirm our findings.

## Data availability statement

The datasets presented in this study can be found in online repositories. The names of the repository/repositories and accession number(s) can be found in the article/**Supplementary Material**.

## Author contributions

XC: Data curation, Investigation, Software, Visualization, Writing – original draft, Writing – review & editing. XH: Software, Writing – original draft. LX: Methodology, Writing – original draft. XL: Software, Writing – original draft, Writing – review & editing.
