# Supplementary material for: A bidirectional causal relationship study between mental disorders and male and female infertility
Source: Front Psychiatry. 2024 Apr 18;15:1378224. doi: 10.3389/fpsyt.2024.1378224 (PMC11064171; doi:10.3389/fpsyt.2024.1378224)
Supplement: Supplementary file 1 [file DataSheet_1.docx]

**Supplementary Material**

**Supplementary Table 1.** Mendelian randomization analysis of causal relationship between mental disorders and infertility.

**Supplementary Table 2.** Mendelian randomization analysis of causal relationship between infertility and mental disorders.

**Supplementary Table 3.** Heterogeneity and pleiotropy between mental disorders and infertility assessed using different methods.

**Supplementary Table 4.** Heterogeneity and pleiotropy between infertility and mental disorders assessed using different methods.

**Supplementary Figure 1.A.** Scatter plot and leave-one-out plot of the causal relationship between attention deficit hyperactivity disorder and male infertility. **1.B.** Scatter plot and leave-one-out plot of the causal relationship between mood disorders and male infertility. **1.C.** Scatter plot and leave-one-out plot of the causal relationship between obsessive-compulsive disorder and male infertility.

**Supplementary Figure 2.A.** Scatter plot and leave-one-out plot of the causal relationship between anorexia nervosa and female infertility. **2.B.** Scatter plot and leave-one-out plot of the causal relationship between attention deficit hyperactivity disorder and female infertility. **2.C.** Scatter plot and leave-one-out plot of the causal relationship between major depressive disorder and female infertility.

**Supplementary Figure 3.A.** Radial Mendelian randomization analysis of the causal relationship between male infertility and schizophrenia. **3.B.** Radial Mendelian randomization analysis of the causal relationship between female infertility and insomnia.

**Supplementary Figure 4.** Scatter plot and leave-one-out plot of the causal relationship between female infertility and bipolar disorder.

**Supplementary Figure 5.A.** Adjusted causal effects of cigarettes per day, alcoholic drinks per week, and attention deficit hyperactivity disorder on the risk of male infertility by multivariable Mendelian randomization analysis. **5.B.** Adjusted causal effects of cigarettes per day, alcoholic drinks per week, and female infertility on the risk of bipolar disorder by multivariable Mendelian randomization analysis.

**Supplementary Table 1.** Mendelian randomization analysis of causal relationship between mental disorders and infertility.

| Exposure | Outcome | SNPs | Methods | OR (95% CI) | P value |
| --- | --- | --- | --- | --- | --- |
| Alzheimer's disease | Male infertility | 46 | MR Egger | 0.8831(0.7305–1.0676) | 0.2068 |
|  |  |  | Weighted median | 0.9026(0.7396–1.1016) | 0.3137 |
|  |  |  | Inverse variance weighted | 0.9167(0.8022–1.0475) | 0.2012 |
| Anorexia nervosa | Male infertility | 14 | MR Egger | 0.8124(0.4754–1.3883) | 0.4633 |
|  |  |  | Weighted median | 0.9101(0.6746–1.2279) | 0.5377 |
|  |  |  | Inverse variance weighted | 0.9358(0.7501–1.1675) | 0.5569 |
| Anxiety | Male infertility | 7 | MR Egger | 3.7144e+11(6.2586e-75–2.2044e+97) | 0.8020 |
|  |  |  | Weighted median | 1.1493e+10(8.6427e-14–1.5284e+33) | 0.3938 |
|  |  |  | Inverse variance weighted | 2.7752e+01(1.9804e-19–3.8888e+21) | 0.8883 |
| Attention deficit hyperactivity disorder | Male infertility | 30 | MR Egger | 0.8312(0.3425–2.0173) | 0.6860 |
|  |  |  | Weighted median | 1.2643(0.9072–1.7620) | 0.1661 |
|  |  |  | Inverse variance weighted | **1.3921(1.0943–1.7709)** | **0.0071** |
| Autism spectrum disorder | Male infertility | 34 | MR Egger | 0.8094(0.3382–1.9370) | 0.6384 |
|  |  |  | Weighted median | 1.0508(0.7107–1.5537) | 0.8036 |
|  |  |  | Inverse variance weighted | 0.9971(0.7489–1.3274) | 0.9841 |
| Bipolar disorder | Male infertility | 30 | MR Egger | 8.0262e-06(4.1559e-22–1.5500e+11) | 0.5446 |
|  |  |  | Weighted median | 2.7277e-06(3.7034e-22–2.0090e+10) | 0.4918 |
|  |  |  | Inverse variance weighted | 5.3410e-01(1.5446e-11–1.8467e+10) | 0.9595 |
| Depression | Male infertility | 32 | MR Egger | 5.7913e-09(6.7463e-19–4.9715e+01) | 0.1149 |
|  |  |  | Weighted median | 1.6842(9.7535e-06–2.9082e+05) | 0.9324 |
|  |  |  | Inverse variance weighted | 2.6478e-02(7.2383e-06–9.6857e+01) | 0.3856 |
| Epilepsy | Male infertility | 9 | MR Egger | 6.6778e-52(2.1801e-143–2.0454e+40) | 0.3091 |
|  |  |  | Weighted median | 5.1866e+02(4.5016e-20–5.9758e+24) | 0.8094 |
|  |  |  | Inverse variance weighted | 3.0519e-10(1.6313e-27–5.7094e+07) | 0.2802 |
| Insomnia | Male infertility | 174 | MR Egger | 0.1374(0.0035–5.3363) | 0.2894 |
|  |  |  | Weighted median | 0.3890(0.0676–2.2391) | 0.2904 |
|  |  |  | Inverse variance weighted | 0.8814(0.2908–2.6709) | 0.8234 |
| Major depressive disorder | Male infertility | 55 | MR Egger | 1.0293(0.5908–1.7931) | 0.9191 |
|  |  |  | Weighted median | 0.8879(0.5076–1.5531) | 0.6768 |
|  |  |  | Inverse variance weighted | 0.7908(0.5764–1.0851) | 0.1460 |
| Mood disorders | Male infertility | 35 | MR Egger | 1.4302(0.5584–3.6634) | 0.4612 |
|  |  |  | Weighted median | 1.5424(0.9652–2.4648) | 0.0700 |
|  |  |  | Inverse variance weighted | **1.4497(1.0093–2.0823)** | **0.0444** |
| Obsessive-compulsive disorder | Male infertility | 13 | MR Egger | 0.9254(0.6771–1.2646) | 0.6370 |
|  |  |  | Weighted median | 0.9001(0.7496–1.0809) | 0.2599 |
|  |  |  | Inverse variance weighted | **0.8208(0.7146–0.9429)** | **0.0052** |
| Parkinson's disease | Male infertility | 50 | MR Egger | 1.2561(0.9745–1.6192) | 0.0847 |
|  |  |  | Weighted median | 1.1562(0.9455–1.4139) | 0.1571 |
|  |  |  | Inverse variance weighted | 1.0844(0.9523–1.2348) | 0.2214 |
| Schizophrenia | Male infertility | 230 | MR Egger | 1.6788(1.0725–2.6277) | 0.0243 |
|  |  |  | Weighted median | 1.1365(0.9537–1.3545) | 0.1525 |
|  |  |  | Inverse variance weighted | 1.0589(0.9400–1.1928) | 0.3457 |
| Stroke | Male infertility | 4 | MR Egger | 1.4001e+18(1.6137e-76–1.2148e+112) | 0.7413 |
|  |  |  | Weighted median | 2.4493e+07(1.3633e-15–4.4003e+29) | 0.5151 |
|  |  |  | Inverse variance weighted | 3.9253e+07(2.2889e-13–6.7316e+27) | 0.4619 |
| Alzheimer's disease | Female infertility | 46 | MR Egger | 1.0705(0.9978–1.1486) | 0.0649 |
|  |  |  | Weighted median | 1.0319(0.9659–1.1023) | 0.3510 |
|  |  |  | Inverse variance weighted | 1.0417(0.9911–1.0949) | 0.1074 |
| Anorexia nervosa | Female infertility | 14 | MR Egger | 1.0567(0.8652–1.2907) | 0.5995 |
|  |  |  | Weighted median | 1.0855(0.9741–1.2096) | 0.1376 |
|  |  |  | Inverse variance weighted | **1.0898(1.0070–1.1794)** | **0.0329** |
| Anxiety | Female infertility | 7 | MR Egger | 106828.1172(5.6122e-19–2.0334e+28) | 0.6896 |
|  |  |  | Weighted median | 81.6298(8.7907e-06–7.5800e+08) | 0.5907 |
|  |  |  | Inverse variance weighted | 33.3756(1.0824e-04–1.0291e+07) | 0.5864 |
| Attention deficit hyperactivity disorder | Female infertility | 30 | MR Egger | 1.1837(0.8379–1.6722) | 0.3473 |
|  |  |  | Weighted median | 1.1307(1.0036–1.2739) | 0.0436 |
|  |  |  | Inverse variance weighted | **1.1013(1.0041–1.2079)** | **0.0406** |
| Autism spectrum disorder | Female infertility | 34 | MR Egger | 1.0335(0.7611–1.4033) | 0.8342 |
|  |  |  | Weighted median | 1.0473(0.9102–1.2052) | 0.5179 |
|  |  |  | Inverse variance weighted | 1.0446(0.9462–1.1533) | 0.3865 |
| Bipolar disorder | Female infertility | 30 | MR Egger | 5.1461e-01(1.5889e-07–1666736.93) | 0.9314 |
|  |  |  | Weighted median | 1.2059e+02(5.2274e-04–27822964.43) | 0.4468 |
|  |  |  | Inverse variance weighted | 6.7766(4.8450e-04–94784.04) | 0.6944 |
| Depression | Female infertility | 32 | MR Egger | 2.9433(1.1937e-03–7257.4403) | 0.7883 |
|  |  |  | Weighted median | 5.0294(1.0386e-01–243.5324) | 0.4145 |
|  |  |  | Inverse variance weighted | 2.2269(1.3542e-01–36.6178) | 0.5751 |
| Epilepsy | Female infertility | 9 | MR Egger | 272.485(1.1255e-29–6.5968e+33) | 0.8834 |
|  |  |  | Weighted median | 113495.838(4.0874e-03–3.1514e+12) | 0.1831 |
|  |  |  | Inverse variance weighted | 3501.325(4.2895e-03–2.8579e+09) | 0.2399 |
| Insomnia | Female infertility | 174 | MR Egger | 2.6395(0.7624–9.1387) | 0.1275 |
|  |  |  | Weighted median | 1.4111(0.7760–2.5659) | 0.2588 |
|  |  |  | Inverse variance weighted | 1.4010(0.9604–2.0438) | 0.0800 |
| Major depressive disorder | Female infertility | 55 | MR Egger | 1.1085(0.9080–1.3532) | 0.3168 |
|  |  |  | Weighted median | 1.1165(0.9315–1.3384) | 0.2332 |
|  |  |  | Inverse variance weighted | **1.1423(1.0213–1.2778)** | **0.0199** |
| Mood disorders | Female infertility | 35 | MR Egger | 1.1183(0.8121–1.5400) | 0.4978 |
|  |  |  | Weighted median | 0.9995(0.8368–1.1938) | 0.9958 |
|  |  |  | Inverse variance weighted | 1.0021(0.8857–1.1339) | 0.9725 |
| Obsessive-compulsive disorder | Female infertility | 13 | MR Egger | 1.0171(0.9139–1.1320) | 0.7621 |
|  |  |  | Weighted median | 0.9948(0.9352–1.0581) | 0.8686 |
|  |  |  | Inverse variance weighted | 0.9992(0.9529–1.0478) | 0.9748 |
| Parkinson's disease | Female infertility | 50 | MR Egger | 0.9195(0.8422–1.0040) | 0.0677 |
|  |  |  | Weighted median | 0.9777(0.9140–1.0458) | 0.5119 |
|  |  |  | Inverse variance weighted | 0.9629(0.9209–1.0069) | 0.0976 |
| Schizophrenia | Female infertility | 230 | MR Egger | 0.9335(0.8013–1.0875) | 0.3781 |
|  |  |  | Weighted median | 1.0260(0.9658–1.0900) | 0.4050 |
|  |  |  | Inverse variance weighted | 0.9956(0.9560–1.0369) | 0.8338 |
| Stroke | Female infertility | 4 | MR Egger | 1.3207e-21(4.6091e-63–3.7845e+20) | 0.4276 |
|  |  |  | Weighted median | 1.0609e-03(5.6783e-12–1.9821e+05) | 0.4809 |
|  |  |  | Inverse variance weighted | 1.7100e-03(4.6326e-12–6.3121e+05) | 0.5267 |

**Supplementary Table 2.** Mendelian randomization analysis of causal relationship between infertility and mental disorders.

| Exposure | Outcome | SNPs | Methods | OR (95% CI) | P value |
| --- | --- | --- | --- | --- | --- |
| Male infertility | Alzheimer's disease | 9 | MR Egger | 0.9701(0.9225–1.0200) | 0.2808 |
|  |  |  | Weighted median | 0.9838(0.9409–1.0285) | 0.4722 |
|  |  |  | Inverse variance weighted | 0.9852(0.9518–1.0197) | 0.3966 |
| Male infertility | Anorexia nervosa | 9 | MR Egger | 1.0366(0.9163–1.1728) | 0.5880 |
|  |  |  | Weighted median | 1.0155(0.9296–1.1093) | 0.7319 |
|  |  |  | Inverse variance weighted | 1.0267(0.9562–1.1024) | 0.4671 |
| Male infertility | Anxiety | 6 | MR Egger | 1.0015(0.9969–1.0061) | 0.5374 |
|  |  |  | Weighted median | 0.9998(0.9989–1.0007) | 0.7340 |
|  |  |  | Inverse variance weighted | 0.9999(0.9992–1.0006) | 0.9317 |
| Male infertility | Attention deficit hyperactivity disorder | 43 | MR Egger | 1.0092(0.9658–1.0546) | 0.6832 |
|  |  |  | Weighted median | 1.0262(0.9960–1.0572) | 0.0890 |
|  |  |  | Inverse variance weighted | 1.0067(0.9855–1.0283) | 0.5353 |
| Male infertility | Autism spectrum disorder | 7 | MR Egger | 1.0528(0.9906–1.1189) | 0.1729 |
|  |  |  | Weighted median | 1.0394(0.9918–1.0893) | 0.1053 |
|  |  |  | Inverse variance weighted | 1.0345(0.9966–1.0738) | 0.0746 |
| Male infertility | Bipolar disorder | 10 | MR Egger | 1.0000(0.9996–1.0003) | 0.9754 |
|  |  |  | Weighted median | 1.0000(0.9997–1.0003) | 0.7564 |
|  |  |  | Inverse variance weighted | 0.9999(0.9997–1.0002) | 0.9803 |
| Male infertility | Depression | 10 | MR Egger | 1.0000(0.9987–1.0014) | 0.8958 |
|  |  |  | Weighted median | 1.0000(0.9988–1.0012) | 0.9235 |
|  |  |  | Inverse variance weighted | 0.9999(0.9990–1.0007) | 0.8458 |
| Male infertility | Epilepsy | 6 | MR Egger | 0.9994(0.9955–1.0034) | 0.8135 |
|  |  |  | Weighted median | 1.0004(0.9996–1.0011) | 0.2776 |
|  |  |  | Inverse variance weighted | 1.0001(0.9996–1.0007) | 0.5034 |
| Male infertility | Insomnia | 12 | MR Egger | 1.0010(0.9972–1.0048) | 0.6035 |
|  |  |  | Weighted median | 1.0008(0.9976–1.0041) | 0.5928 |
|  |  |  | Inverse variance weighted | 1.0004(0.9980–1.0029) | 0.7059 |
| Male infertility | Major depressive disorder | 11 | MR Egger | 0.9873(0.9624–1.0129) | 0.3581 |
|  |  |  | Weighted median | 1.0001(0.9785–1.0221) | 0.9901 |
|  |  |  | Inverse variance weighted | 1.0020(0.9858–1.0184) | 0.8065 |
| Male infertility | Mood disorders | 12 | MR Egger | 1.0105(0.9754–1.0469) | 0.5735 |
|  |  |  | Weighted median | 0.9976(0.9714–1.0245) | 0.8620 |
|  |  |  | Inverse variance weighted | 1.0003(0.9799–1.0212) | 0.9729 |
| Male infertility | Obsessive-compulsive disorder | 12 | MR Egger | 1.0929(0.9377–1.2737) | 0.2818 |
|  |  |  | Weighted median | 0.9959(0.8809–1.1260) | 0.9484 |
|  |  |  | Inverse variance weighted | 0.9975(0.9123–1.0906) | 0.9563 |
| Male infertility | Parkinson's disease | 11 | MR Egger | 0.9944(0.9259–1.0679) | 0.8817 |
|  |  |  | Weighted median | 0.9881(0.9281–1.0519) | 0.7085 |
|  |  |  | Inverse variance weighted | 0.9914(0.9475–1.0373) | 0.7087 |
| Male infertility | Schizophrenia | 10 | MR Egger | 0.9835(0.9227–1.0484) | 0.6251 |
|  |  |  | Weighted median | 0.9845(0.9486–1.0218) | 0.4112 |
|  |  |  | Inverse variance weighted | 0.9673(0.9290–1.0072) | 0.1072 |
| Male infertility | Stroke | 6 | MR Egger | 0.9982(0.9937–1.0027) | 0.4859 |
|  |  |  | Weighted median | 1.0006(0.9997–1.0015) | 0.1773 |
|  |  |  | Inverse variance weighted | 1.0004(0.9997–1.0011) | 0.2138 |
| Female infertility | Alzheimer's disease | 10 | MR Egger | 1.0392(0.8404–1.2851) | 0.7343 |
|  |  |  | Weighted median | 1.0814(0.9457–1.2365) | 0.2522 |
|  |  |  | Inverse variance weighted | 1.0316(0.9320–1.1418) | 0.5470 |
| Female infertility | Anorexia nervosa | 10 | MR Egger | 0.8375(0.5555–1.2627) | 0.4298 |
|  |  |  | Weighted median | 1.2799(0.9669–1.6944) | 0.0845 |
|  |  |  | Inverse variance weighted | 1.1552(0.9388–1.4215) | 0.1726 |
| Female infertility | Anxiety | 6 | MR Egger | 1.0027(0.9961–1.0093) | 0.4621 |
|  |  |  | Weighted median | 1.0017(0.9992–1.0042) | 0.1778 |
|  |  |  | Inverse variance weighted | 1.0016(0.9996–1.0037) | 0.1095 |
| Female infertility | Attention deficit hyperactivity disorder | 6 | MR Egger | 0.8316(0.4065–1.7010) | 0.6483 |
|  |  |  | Weighted median | 0.9651(0.7665–1.2152) | 0.7627 |
|  |  |  | Inverse variance weighted | 0.9451(0.7499–1.1912) | 0.6329 |
| Female infertility | Autism spectrum disorder | 10 | MR Egger | 0.9280(0.7581–1.1361) | 0.4967 |
|  |  |  | Weighted median | 0.9903(0.8634–1.1358) | 0.8892 |
|  |  |  | Inverse variance weighted | 0.9879(0.8953–1.0900) | 0.8085 |
| Female infertility | Bipolar disorder | 10 | MR Egger | 1.0003(0.9986–1.0019) | 0.7477 |
|  |  |  | Weighted median | 1.0010(0.9999–1.0021) | 0.0709 |
|  |  |  | Inverse variance weighted | **1.0009(1.0001–1.0017)** | **0.0281** |
| Female infertility | Depression | 8 | MR Egger | 1.0016(0.9908–1.0125) | 0.7753 |
|  |  |  | Weighted median | 1.0011(0.9961–1.0062) | 0.6440 |
|  |  |  | Inverse variance weighted | 1.0006(0.9969–1.0043) | 0.7233 |
| Female infertility | Epilepsy | 5 | MR Egger | 1.0020(0.9724–1.0326) | 0.9007 |
|  |  |  | Weighted median | 1.0002(0.9979–1.0024) | 0.8570 |
|  |  |  | Inverse variance weighted | 1.0000(0.9981–1.0019) | 0.9913 |
| Female infertility | Insomnia | 10 | MR Egger | 1.0000(0.9682–1.0331) | 0.9928 |
|  |  |  | Weighted median | 1.0034(0.9901–1.0168) | 0.6143 |
|  |  |  | Inverse variance weighted | 1.0046(0.9895–1.0200) | 0.5485 |
| Female infertility | Major depressive disorder | 10 | MR Egger | 0.9180(0.8118–1.0382) | 0.2221 |
|  |  |  | Weighted median | 0.9971(0.9219–1.0784) | 0.9430 |
|  |  |  | Inverse variance weighted | 1.0374(0.9690–1.1107) | 0.2904 |
| Female infertility | Mood disorders | 10 | MR Egger | 0.9725(0.8177–1.1564) | 0.7604 |
|  |  |  | Weighted median | 0.9367(0.8549–1.0262) | 0.1606 |
|  |  |  | Inverse variance weighted | 0.9359(0.8693–1.0077) | 0.0790 |
| Female infertility | Obsessive-compulsive disorder | 10 | MR Egger | 0.7064(0.2432–2.0519) | 0.5408 |
|  |  |  | Weighted median | 0.6220(0.3908–0.9899) | 0.0452 |
|  |  |  | Inverse variance weighted | 0.7009(0.4478–1.0970) | 0.1200 |
| Female infertility | Parkinson's disease | 10 | MR Egger | 1.1197(0.8272–1.5157) | 0.4848 |
|  |  |  | Weighted median | 1.0226(0.8523–1.2268) | 0.8099 |
|  |  |  | Inverse variance weighted | 0.9737(0.8478–1.1183) | 0.7066 |
| Female infertility | Schizophrenia | 10 | MR Egger | 1.0474(0.9112–1.2039) | 0.5325 |
|  |  |  | Weighted median | 0.9933(0.9029–1.0928) | 0.8908 |
|  |  |  | Inverse variance weighted | 1.0085(0.9403–1.0816) | 0.8125 |
| Female infertility | Stroke | 6 | MR Egger | 1.0053(0.9988–1.0117) | 0.1810 |
|  |  |  | Weighted median | 0.9991(0.9962–1.0020) | 0.5683 |
|  |  |  | Inverse variance weighted | 0.9986(0.9962–1.0010) | 0.2852 |

**Supplementary Table 3.** Heterogeneity and pleiotropy between mental disorders and infertility assessed using different methods.

| Exposure | Outcome | Methods | Heterogeneity | | Pleiotropy | | | MR-PRESSO |
| --- | --- | --- | --- | --- | --- | --- | --- | --- |
|  |  |  | Q | P value | Egger_intercept | SE | P value | P value |
| Alzheimer's disease | Male infertility | MR Egger | 39.1534 | 0.4178 | 0.0098 | 0.0179 | 0.5872 | 0.6180 |
|  |  | IVW | 39.4622 | 0.4492 |  |  |  |  |
| Anorexia nervosa | Male infertility | MR Egger | 9.7313 | 0.5547 | 0.0343 | 0.0605 | 0.5814 | 0.7020 |
|  |  | IVW | 10.0539 | 0.6112 |  |  |  |  |
| Anxiety | Male infertility | MR Egger | 10.3003 | 0.0671 | -0.0349 | 0.1459 | 0.8203 | 0.1200 |
|  |  | IVW | 10.4184 | 0.1081 |  |  |  |  |
| Attention deficit hyperactivity disorder | Male infertility | MR Egger | 22.7563 | 0.6980 | 0.0535 | 0.0451 | 0.2465 | 0.6530 |
|  |  | IVW | 24.1594 | 0.6730 |  |  |  |  |
| Autism spectrum disorder | Male infertility | MR Egger | 17.6495 | 0.9511 | 0.0191 | 0.0387 | 0.6237 | 0.9480 |
|  |  | IVW | 17.8954 | 0.9602 |  |  |  |  |
| Bipolar disorder | Male infertility | MR Egger | 20.7091 | 0.8372 | 0.0249 | 0.0327 | 0.4528 | 0.8570 |
|  |  | IVW | 21.2888 | 0.8484 |  |  |  |  |
| Depression | Male infertility | MR Egger | 26.3944 | 0.6043 | 0.0510 | 0.0362 | 0.1698 | 0.5990 |
|  |  | IVW | 28.3762 | 0.5505 |  |  |  |  |
| Epilepsy | Male infertility | MR Egger | 5.3646 | 0.6155 | 0.1071 | 0.1178 | 0.3936 | 0.6140 |
|  |  | IVW | 6.1906 | 0.6258 |  |  |  |  |
| Insomnia | Male infertility | MR Egger | 181.9140 | 0.1131 | 0.0187 | 0.0179 | 0.2978 | 0.1360 |
|  |  | IVW | 183.1541 | 0.1114 |  |  |  |  |
| Major depressive disorder | Male infertility | MR Egger | 40.0180 | 0.7199 | -0.0199 | 0.0175 | 0.2634 | 0.8590 |
|  |  | IVW | 41.3000 | 0.7067 |  |  |  |  |
| Mood disorders | Male infertility | MR Egger | 22.7400 | 0.9098 | 0.0010 | 0.0357 | 0.9757 | 0.9480 |
|  |  | IVW | 22.7409 | 0.9293 |  |  |  |  |
| Obsessive-compulsive disorder | Male infertility | MR Egger | 8.7120 | 0.5596 | -0.0503 | 0.0599 | 0.4207 | 0.2820 |
|  |  | IVW | 9.4168 | 0.5834 |  |  |  |  |
| Parkinson's disease | Male infertility | MR Egger | 45.6570 | 0.5282 | -0.0269 | 0.0203 | 0.1927 | 0.5490 |
|  |  | IVW | 47.4029 | 0.4972 |  |  |  |  |
| Schizophrenia | Male infertility | MR Egger | 208.4319 | 0.7182 | -0.0341 | 0.0163 | 0.0376 | 0.693 |
|  |  | IVW | 212.8042 | 0.6594 |  |  |  |  |
| Stroke | Male infertility | MR Egger | 0.2109 | 0.8999 | -0.0335 | 0.1487 | 0.8425 | 0.9720 |
|  |  | IVW | 0.2617 | 0.9670 |  |  |  |  |
| Alzheimer's disease | Female infertility | MR Egger | 45.2165 | 0.1960 | -0.0070 | 0.0065 | 0.2890 | 0.3000 |
|  |  | IVW | 46.5922 | 0.1883 |  |  |  |  |
| Anorexia nervosa | Female infertility | MR Egger | 12.9666 | 0.2955 | 0.0074 | 0.0225 | 0.7468 | 0.3640 |
|  |  | IVW | 13.0958 | 0.3621 |  |  |  |  |
| Anxiety | Female infertility | MR Egger | 6.5531 | 0.2560 | -0.0121 | 0.0396 | 0.7724 | 0.3590 |
|  |  | IVW | 6.6753 | 0.3519 |  |  |  |  |
| Attention deficit hyperactivity disorder | Female infertility | MR Egger | 35.1170 | 0.1359 | -0.0074 | 0.0175 | 0.6741 | 0.1870 |
|  |  | IVW | 35.3519 | 0.1598 |  |  |  |  |
| Autism spectrum disorder | Female infertility | MR Egger | 30.9228 | 0.3690 | 0.0009 | 0.0135 | 0.9422 | 0.5580 |
|  |  | IVW | 30.9284 | 0.4189 |  |  |  |  |
| Bipolar disorder | Female infertility | MR Egger | 37.6227 | 0.1057 | 0.0057 | 0.0130 | 0.6622 | 0.1410 |
|  |  | IVW | 37.8846 | 0.1249 |  |  |  |  |
| Depression | Female infertility | MR Egger | 29.1607 | 0.4567 | -0.0009 | 0.0123 | 0.9407 | 0.5590 |
|  |  | IVW | 29.1664 | 0.5088 |  |  |  |  |
| Epilepsy | Female infertility | MR Egger | 2.8858 | 0.8953 | 0.0028 | 0.0403 | 0.9457 | 0.9380 |
|  |  | IVW | 2.8908 | 0.9410 |  |  |  |  |
| Insomnia | Female infertility | MR Egger | 181.1935 | 0.1203 | -0.0063 | 0.0060 | 0.2956 | 0.1760 |
|  |  | IVW | 182.4402 | 0.1185 |  |  |  |  |
| Major depressive disorder | Female infertility | MR Egger | 50.1798 | 0.3112 | 0.0022 | 0.0063 | 0.7213 | 0.4380 |
|  |  | IVW | 50.3203 | 0.3434 |  |  |  |  |
| Mood disorders | Female infertility | MR Egger | 33.3262 | 0.4513 | -0.0088 | 0.0121 | 0.4709 | 0.4800 |
|  |  | IVW | 33.8632 | 0.4743 |  |  |  |  |
| Obsessive-compulsive disorder | Female infertility | MR Egger | 7.2417 | 0.7024 | -0.0074 | 0.0205 | 0.7243 | 0.8480 |
|  |  |  | 7.3733 | 0.7680 |  |  |  |  |
| Parkinson's disease | Female infertility | IVW | 41.8093 | 0.6868 | 0.0083 | 0.0070 | 0.2384 | 0.6940 |
|  |  | MR Egger | 43.2353 | 0.6681 |  |  |  |  |
| Schizophrenia | Female infertility | IVW | 220.9877 | 0.4875 | 0.0047 | 0.0055 | 0.3916 | 0.4280 |
|  |  | MR Egger | 221.7243 | 0.4925 |  |  |  |  |
| Stroke | Female infertility | IVW | 3.3264 | 0.1895 | 0.0574 | 0.0655 | 0.4730 | 0.2670 |
|  |  | MR Egger | 4.6052 | 0.2030 |  |  |  |  |

**Supplementary Table 4.** Heterogeneity and pleiotropy between infertility and mental disorders assessed using different methods.

| Exposure | Outcome | Methods | Heterogeneity | | Pleiotropy | | | MR-PRESSO |
| --- | --- | --- | --- | --- | --- | --- | --- | --- |
|  |  |  | Q | P value | Egger_intercept | SE | P value | P value |
| Male infertility | Alzheimer's disease | MR Egger | 2.1271 | 0.9076 | 0.0122 | 0.0148 | 0.4386 | 0.9050 |
|  |  | IVW | 2.8148 | 0.9015 |  |  |  |  |
| Male infertility | Anorexia nervosa | MR Egger | 7.8314 | 0.2507 | -0.0061 | 0.0314 | 0.8517 | 0.3500 |
|  |  | IVW | 7.8811 | 0.3431 |  |  |  |  |
| Male infertility | Anxiety | MR Egger | 1.6120 | 0.8066 | -0.0005 | 0.0007 | 0.5250 | 0.8540 |
|  |  | IVW | 2.0957 | 0.8357 |  |  |  |  |
| Male infertility | Attention deficit hyperactivity disorder | MR Egger | 33.6307 | 0.5817 | -0.0010 | 0.0084 | 0.8987 | 0.8460 |
|  |  | IVW | 33.6471 | 0.6270 |  |  |  |  |
| Male infertility | Autism spectrum disorder | MR Egger | 3.6012 | 0.4626 | -0.0108 | 0.0152 | 0.5146 | 0.1620 |
|  |  | IVW | 4.1111 | 0.5335 |  |  |  |  |
| Male infertility | Bipolar disorder | MR Egger | 10.1332 | 0.2557 | -6.5037e-06 | 9.7746e-05 | 0.9485 | 0.3390 |
|  |  | IVW | 10.1388 | 0.3393 |  |  |  |  |
| Male infertility | Depression | MR Egger | 8.3650 | 0.3986 | -0.0001 | 0.0003 | 0.7509 | 0.5180 |
|  |  | IVW | 8.4779 | 0.4867 |  |  |  |  |
| Male infertility | Epilepsy | MR Egger | 4.8747 | 0.3003 | 0.0002 | 0.0006 | 0.7434 | 0.4580 |
|  |  | IVW | 5.0246 | 0.4128 |  |  |  |  |
| Male infertility | Insomnia | MR Egger | 11.6099 | 0.3120 | -0.0004 | 0.0010 | 0.7010 | 0.4420 |
|  |  | IVW | 11.7912 | 0.3795 |  |  |  |  |
| Male infertility | Major depressive disorder | MR Egger | 3.1356 | 0.9255 | 0.0100 | 0.0068 | 0.1818 | 0.4890 |
|  |  | IVW | 5.2737 | 0.8098 |  |  |  |  |
| Male infertility | Mood disorders | MR Egger | 5.3047 | 0.8699 | -0.0056 | 0.0081 | 0.5045 | 0.9000 |
|  |  | IVW | 5.7839 | 0.8873 |  |  |  |  |
| Male infertility | Obsessive-compulsive disorder | MR Egger | 5.7189 | 0.8382 | -0.0505 | 0.0351 | 0.1807 | 0.7460 |
|  |  | IVW | 7.7896 | 0.7320 |  |  |  |  |
| Male infertility | Parkinson's disease | MR Egger | 10.7247 | 0.2950 | -0.0020 | 0.0185 | 0.9127 | 0.3800 |
|  |  | IVW | 10.7398 | 0.3781 |  |  |  |  |
| Male infertility | Schizophrenia | MR Egger | 27.2452 | 0.0006 | -0.0110 | 0.0164 | 0.5191 | 0.0020 |
|  |  | IVW | 28.7938 | 0.0007 |  |  |  |  |
| Male infertility | Stroke | MR Egger | 2.4072 | 0.6613 | 0.0006 | 0.0007 | 0.3859 | 0.6500 |
|  |  | IVW | 3.3526 | 0.6457 |  |  |  |  |
| Female infertility | Alzheimer's disease | MR Egger | 5.2456 | 0.5127 | -0.0011 | 0.0153 | 0.9410 | 0.7560 |
|  |  | IVW | 5.2515 | 0.6292 |  |  |  |  |
| Female infertility | Anorexia nervosa | MR Egger | 4.3705 | 0.6266 | 0.0529 | 0.0300 | 0.1288 | 0.4990 |
|  |  | IVW | 7.4687 | 0.3817 |  |  |  |  |
| Female infertility | Anxiety | MR Egger | 1.6570 | 0.7984 | -0.0001 | 0.0004 | 0.7583 | 0.8910 |
|  |  | IVW | 1.7656 | 0.8805 |  |  |  |  |
| Female infertility | Attention deficit hyperactivity disorder | MR Egger | 7.5849 | 0.0554 | 0.0192 | 0.0511 | 0.7315 | 0.1250 |
|  |  | IVW | 7.9433 | 0.0936 |  |  |  |  |
| Female infertility | Autism spectrum disorder | MR Egger | 4.9088 | 0.5555 | 0.0102 | 0.0147 | 0.5143 | 0.6670 |
|  |  | IVW | 5.3887 | 0.6126 |  |  |  |  |
| Female infertility | Bipolar disorder | MR Egger | 6.7760 | 0.5609 | 0.0001 | 0.0001 | 0.4141 | 0.6030 |
|  |  | IVW | 7.5179 | 0.5833 |  |  |  |  |
| Female infertility | Depression | MR Egger | 7.5004 | 0.2770 | -0.0001 | 0.0007 | 0.8545 | 0.3500 |
|  |  | IVW | 7.5461 | 0.3743 |  |  |  |  |
| Female infertility | Epilepsy | MR Egger | 0.1807 | 0.9806 | -0.0002 | 0.0015 | 0.9010 | 0.9960 |
|  |  | IVW | 0.1990 | 0.9953 |  |  |  |  |
| Female infertility | Insomnia | MR Egger | 22.8072 | 0.0036 | 0.0007 | 0.0025 | 0.7629 | 0.007 |
|  |  | IVW | 23.0848 | 0.0060 |  |  |  |  |
| Female infertility | Major depressive disorder | MR Egger | 5.0524 | 0.5371 | 0.0191 | 0.0087 | 0.0698 | 0.3670 |
|  |  | IVW | 9.9035 | 0.1941 |  |  |  |  |
| Female infertility | Mood disorders | MR Egger | 9.9716 | 0.2670 | -0.0060 | 0.0124 | 0.6417 | 0.3910 |
|  |  | IVW | 10.2629 | 0.3296 |  |  |  |  |
| Female infertility | Obsessive-compulsive disorder | MR Egger | 20.3922 | 0.0089 | -0.0012 | 0.0766 | 0.9876 | 0.013 |
|  |  |  | 20.3928 | 0.0156 |  |  |  |  |
| Female infertility | Parkinson's disease | IVW | 6.4408 | 0.5979 | -0.0226 | 0.0222 | 0.3389 | 0.6280 |
|  |  | MR Egger | 7.4749 | 0.5877 |  |  |  |  |
| Female infertility | Schizophrenia | IVW | 7.9033 | 0.4429 | -0.0066 | 0.0107 | 0.5545 | 0.5360 |
|  |  | MR Egger | 8.2836 | 0.5058 |  |  |  |  |
| Female infertility | Stroke | IVW | 2.6810 | 0.6125 | -0.0008 | 0.0003 | 0.1009 | 0.2370 |
|  |  | MR Egger | 7.1908 | 0.2068 |  |  |  |  |


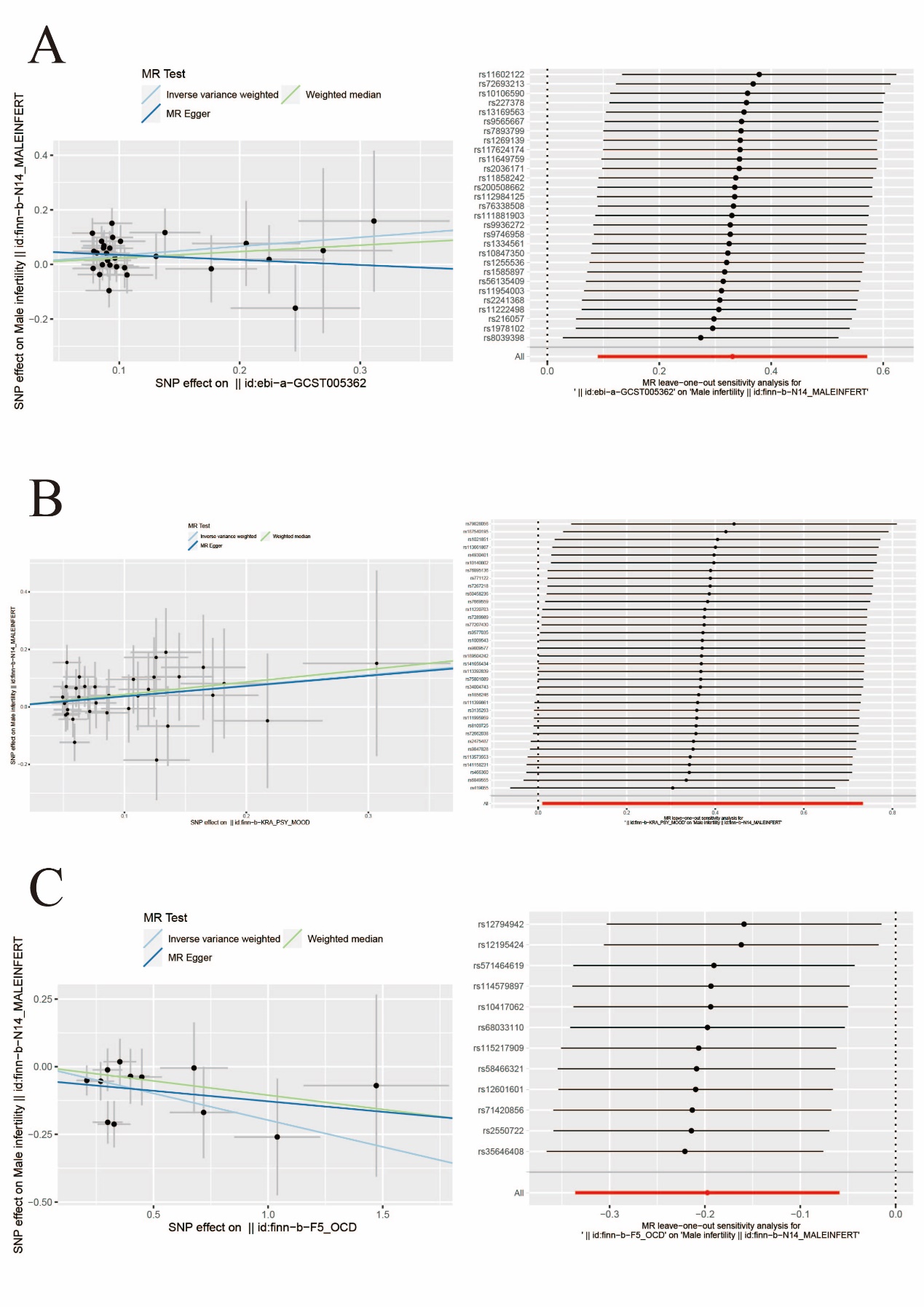


**Supplementary Figure 1.A.** Scatter plot and leave-one-out plot of the causal relationship between attention deficit hyperactivity disorder and male infertility. **1.B.** Scatter plot and leave-one-out plot of the causal relationship between mood disorders and male infertility. **1.C.** Scatter plot and leave-one-out plot of the causal relationship between obsessive-compulsive disorder and male infertility.


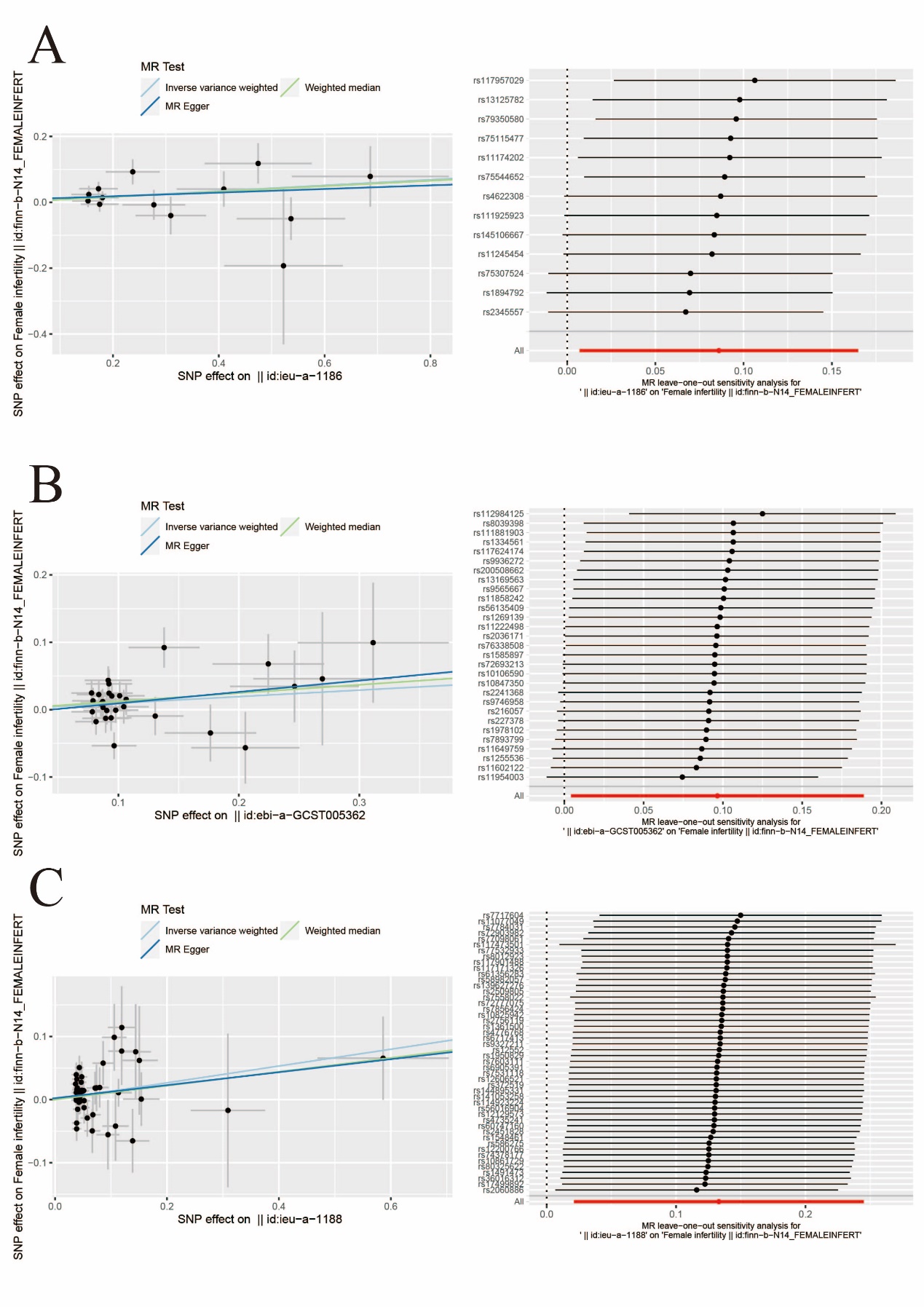


**Supplementary Figure 2.A.** Scatter plot and leave-one-out plot of the causal relationship between anorexia nervosa and female infertility. **2.B.** Scatter plot and leave-one-out plot of the causal relationship between attention deficit hyperactivity disorder and female infertility. **2.C.** Scatter plot and leave-one-out plot of the causal relationship between major depressive disorder and female infertility.


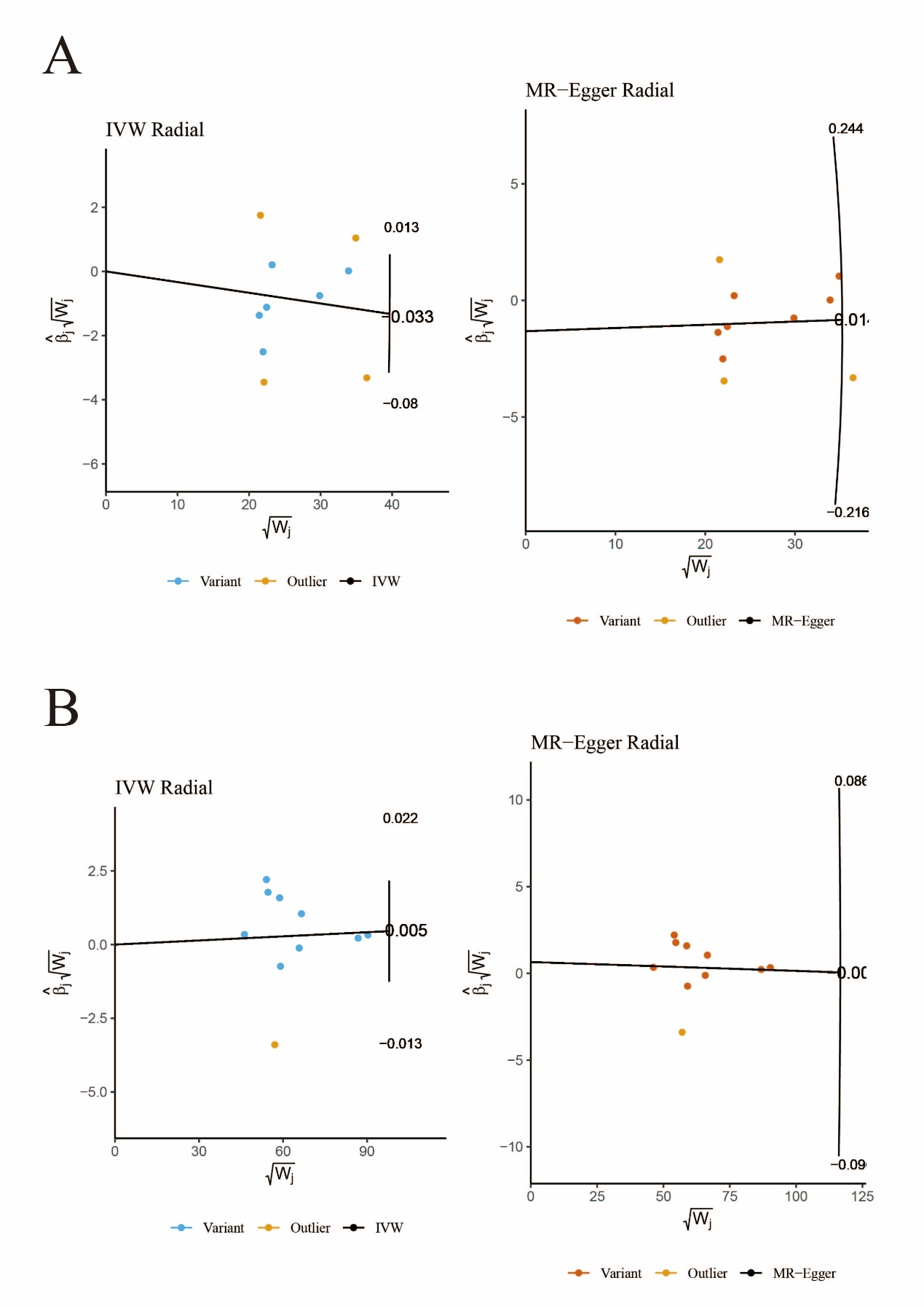


**Supplementary Figure 3.A.** Radial Mendelian randomization analysis of the causal relationship between male infertility and schizophrenia. **3.B.** Radial Mendelian randomization analysis of the causal relationship between female infertility and insomnia.


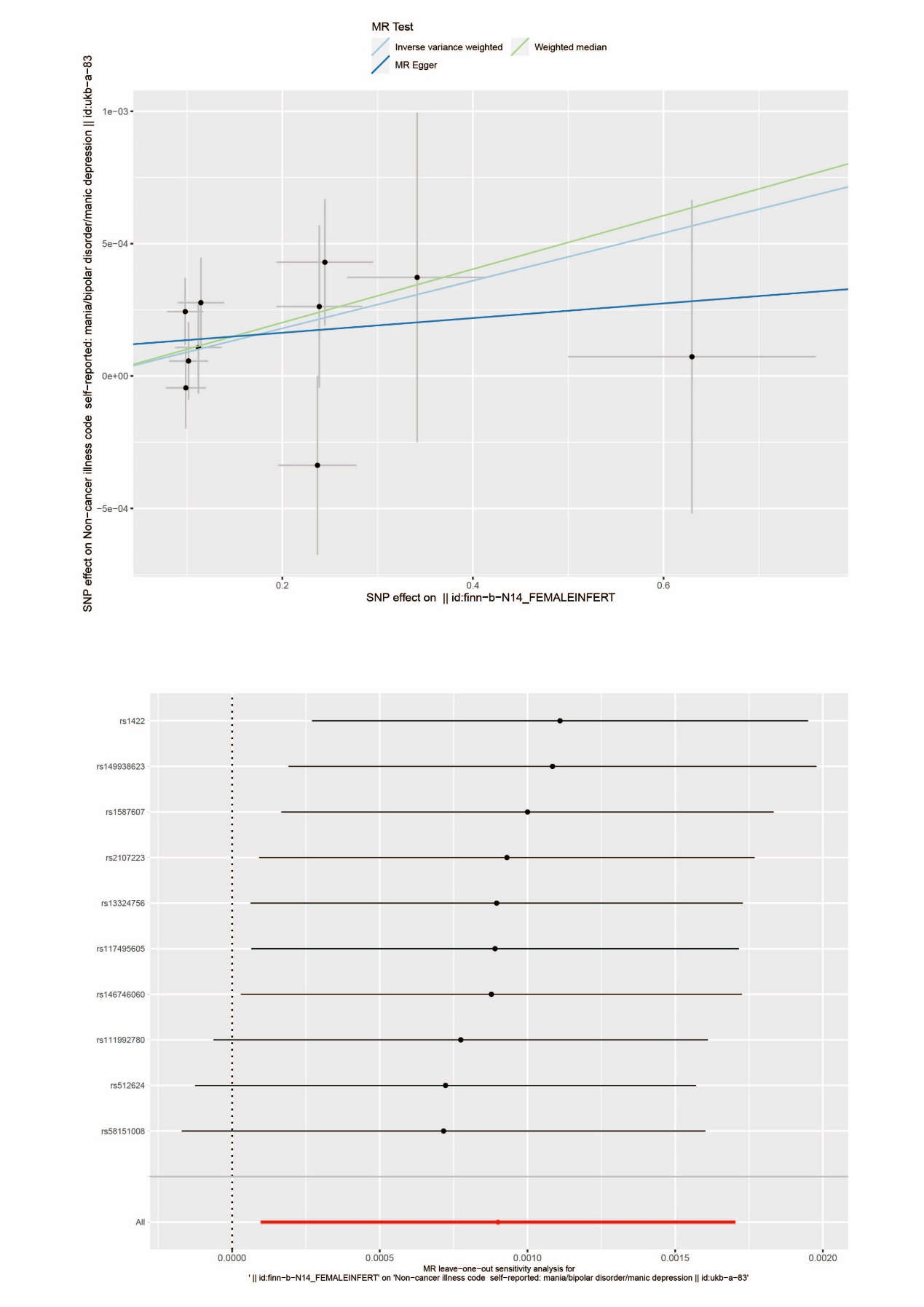


**Supplementary Figure 4.** Scatter plot and leave-one-out plot of the causal relationship between female infertility and bipolar disorder.


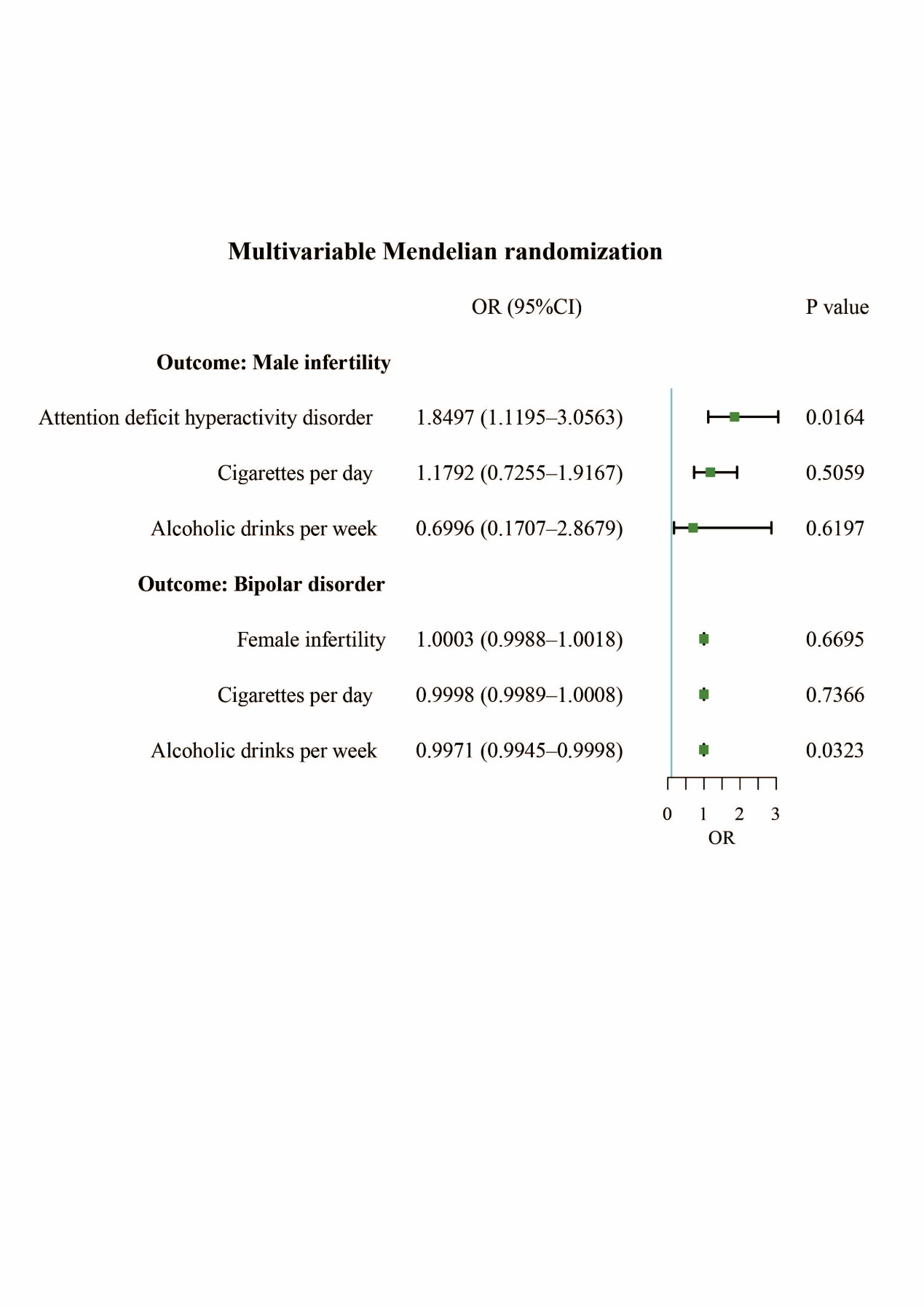


**Supplementary Figure 5.A.** Adjusted causal effects of cigarettes per day, alcoholic drinks per week, and attention deficit hyperactivity disorder on the risk of male infertility by multivariable Mendelian randomization analysis. **5.B.** Adjusted causal effects of cigarettes per day, alcoholic drinks per week, and female infertility on the risk of bipolar disorder by multivariable Mendelian randomization analysis.
